# Genomic characterization of SARS-CoV-2 in Guinea, West Africa

**DOI:** 10.1371/journal.pone.0299082

**Published:** 2024-03-06

**Authors:** Mamadou Saliou Sow, Josue Togo, Lacy M. Simons, Souleymane Taran Diallo, Mohamed Lamine Magassouba, Mamadou Bhoye Keita, Anou Moise Somboro, Youssouf Coulibaly, Egon A. Ozer, Judd F. Hultquist, Robert Leo Murphy, Almoustapha Issiaka Maiga, Mamoudou Maiga, Ramon Lorenzo-Redondo

**Affiliations:** 1 Service de Maladie Infectieuse du Centre Hospitalier de Donka, Conakry, Guinée; 2 University Clinical Research Center, University of Sciences, Techniques and Technologies of Bamako (USTTB), Bamako, Mali; 3 Département de Biologie médicale, Centre Hospitalier Universitaire Gabriel Toure, Bamako, Mali; 4 Division of Infectious Diseases, Department of Medicine, Northwestern University Feinberg School of Medicine, Chicago, IL, United States of America; 5 Center for Pathogen Genomics and Microbial Evolution, Northwestern University Havey Institute for Global Health, Chicago, IL, United States of America; 6 Département de laboratoire, Institut National de la Santé Publique, Conakry, Guinée; 7 Département de biologie, Université Gamal Abdel Nasser de Conakry, Conakry, Guinée; 8 Antimicrobial Research Unit, College of Health Sciences, University of KwaZulu-Natal, Durban, South Africa; 9 Institute for Global Health, Northwestern University, Chicago, IL, United States of America; Federal Medical Centre Abeokuta, NIGERIA

## Abstract

SARS-CoV-2 has claimed several million lives since its emergence in late 2019. The ongoing evolution of the virus has resulted in the periodic emergence of new viral variants with distinct fitness advantages, including enhanced transmission and immune escape. While several SARS-CoV-2 variants of concern trace their origins back to the African continent—including Beta, Eta, and Omicron–most countries in Africa remain under-sampled in global genomic surveillance efforts. In an effort to begin filling these knowledge gaps, we conducted retrospective viral genomic surveillance in Guinea from October 2020 to August 2021. We found that SARS-CoV-2 clades 20A, 20B, and 20C dominated throughout 2020 until the coincident emergence of the Alpha and Eta variants of concern in January 2021. The Alpha variant remained dominant throughout early 2021 until the arrival of the Delta variant in July. Surprisingly, despite the small sample size of our study, we also found the persistence of the early SARS-CoV-2 clade 19B as late as April 2021. Together, these data help fill in our understanding of the SARS-CoV-2 population dynamics in West Africa early in the COVID-19 pandemic.

## Introduction

After its emergence in Wuhan, China in late 2019, SARS-CoV-2 (Severe Acute Respiratory Syndrome Coronavirus 2), the causative agent of COVID-19 (Coronavirus disease 2019), has spread across the world causing massive morbidity and mortality. As of February 2023, over 750 million confirmed cases have been reported [1], resulting in more than 6.5 million deaths, with over 400,000 new cases still being reported each day [1, 2].

Africa has observed a relatively low proportion of confirmed cases and deaths compared to the other continents. While Africa contains nearly 17% of the global population, it accounts for less than 3% of reported COVID-19 related deaths worldwide (only 175,289 deaths as of February 2023) [1]. In Guinea, a low-income West African country, the first cases of COVID-19 were reported in March 2020 with only 38,267 confirmed cases and 467 (1.2%) deaths as of February 2023 [1]. Despite numerous studies [3–11], it is still unclear if the relatively low incidence is due to underreporting or lack of public health surveillance, the circulation of variants with lower transmission or virulence, climate and other environmental factors that alter viral spread or pathogenesis, underlying regional immunity to coronaviruses, or a combination of these factors.

The ongoing evolution of SARS-CoV-2 has resulted in the periodic emergence and spread of variants with enhanced viral fitness [12–15]. These are grouped into four classes (variants of interest, variants of concern, variants of high consequence, and variants being monitored) by the US Centers for Disease Control and Prevention (CDC) [15] and the World Health Organization (WHO), [12] based on a variant’s transmissibility, virulence, and responsiveness to treatment and prevention measures. Despite the fact that numerous variants of concern (VOCs) have emerged from the African continent—including Beta, Eta, and Omicron–genomic surveillance for the detection and monitoring of variants in most African countries remains low. Only 1% of all deposited SARS-CoV-2 genome sequences in the GISAID public repository are from Africa and a majority of these are from South Africa, which was uniquely positioned to perform genomic surveillance in the region. Lower income countries in West Africa are particularly underrepresented in these databases, especially early in the pandemic.

To begin to address this knowledge gap, we endeavored to perform retrospective genomic surveillance using banked clinical specimens from Guinea early in the COVID-19 pandemic. Here, we report on the population dynamics of SARS-CoV-2 in Guinea in 2020 and 2021, which may offer new insights into the evolution of the virus in West Africa.

## Materials and methods

### Study population and specimen collection

We conducted a cross-sectional study to describe the virological profile of SARS-CoV-2 in Guinea in sub-Saharan Africa. Donka University Hospital was the reference center for data collection and although most of the data came from the Conakry administrative region, we collected data from the entire country of Guinea. The research protocol has been approved by the National Health Research Ethics Committee (CNRES) of the Republic of Guinea with the reference number: No_112/CNERS/20. Briefly, consented individuals were distributed a survey questionnaire via RedCap for the self-reporting of socio-demographic characteristics. Each participant subsequently underwent a physical and clinical exam by a physician for the collection of clinical data and nasopharyngeal (NP) swabs. Two NP swabs were collected in Phosphate-Buffered Saline (PBS) from each participant. One was used to conduct a quantitative reverse transcriptase polymerase chain reaction (qRT-PCR CDC 2019-nCoV RT-PCR Diagnostic Panel) test for SARS-CoV-2 diagnosis on-site. All participants that tested positive for SARS-CoV-2 (cycle threshold value <35) between October 2020 and August 2021 were included in this study (n = 99 participants total). The second NP swab specimen was sent to the Center for Pathogen Genomics and Microbial Evolution at Northwestern University for viral whole genome sequencing.

### Viral load determination and cDNA synthesis

Viral RNA was extracted from nasopharyngeal specimens utilizing the QIAamp Viral RNA Minikit (Qiagen). Confirmatory testing for SARS-CoV-2 was performed by qRT-PCR with the CDC 2019-nCoV RT-PCR Diagnostic Panel utilizing N1 and RNase P probes as previously described [IDT, cat. no. 10006713]. RNA samples of sufficient quality (RNaseP control, Ct value <35) and with sufficient copies of the viral genome for sequencing (N1, Ct value <32) were reverse transcribed into complementary DNA (cDNA) [16]. cDNA synthesis was performed with the SuperScript IV First Strand Synthesis Kit (Thermo) using random hexamer primers according to the manufacturer’s specifications. Direct amplification of the viral genome cDNA was performed in multiplexed PCR reactions to generate ~400 base pair amplicons tiled across the genome. The multiplex primer set, comprised of two non-overlapping primer pools, was created using Primal Scheme and provided by the Artic Network (version 3 release). PCR amplification was carried out using Q5 Hot Start HF Taq Polymerase (NEB) with 5μl of cDNA in a 25μl reaction volume. A two-step PCR program was used with an initial step of 98°C for 30 seconds, then 35 cycles of 98°C for 15 seconds followed by five minutes at 65°C. Separate reactions were carried out for each primer pool and validated by agarose gel electrophoresis.

### Sequencing library preparation, Illumina sequencing, and genome assembly

Uniquely barcoded samples were pooled, and sequencing libraries were prepared using the SeqWell plexWell 384 kit per manufacturer’s instructions. The pooled library was denatured and loaded onto a MiSeq v2 500 cycle flow cell (Illumina). Reads barcodes, adapters and low-quality sequence were trimmed using Trimmomatic v0.39 then aligned to the reference SARS-CoV-2 genome sequence MN908947.3 using BWA version 0.7.17. Consensus sequences were determined using iVar v1.2.2 [17] with a minimum alignment depth of 10 reads, minimum base quality of 20, and a consensus frequency threshold of 0 (*i*.*e*., majority base as the consensus). Consensus sequences with ≥ 10% missing bases were discarded. Of the 99 specimens processed, a majority (n = 80) failed to yield a satisfactory consensus sequence due to insufficient genetic material, insufficient purity after barcoding, or inadequate read coverage after sequencing; these samples were excluded from further analysis. The complete SARS-CoV-2 genomes for the other 19 specimens were deposited in the public GISAID database (Table 1) and subjected to phylogenetic analysis.

**Table 1:** GISAID accession numbers associated with this study.

Count	Isolation Date	Location	GISAID Accession Number	Pango Lineage	NextClade
1	2020	Africa / Guinea	TBD	B.1	20A
2	10/5/20	Africa / Guinea / Conakry	EPI_ISL_4274017	B.1	20A
3	9/29/20	Africa / Guinea / Conakry	EPI_ISL_4274019	B.1.1.1	20D
4	9/28/20	Africa / Guinea / Conakry	EPI_ISL_4274021	B.1	20A
5	10/15/20	Africa / Guinea / Conakry	EPI_ISL_4274022	B.1	20A
6	12/18/20	Africa / Guinea / Conakry	EPI_ISL_4274024	B.1	20A
7	2020	Africa / Guinea	TBD	A.27	19B
8	12/17/20	Africa / Guinea / Conakry	EPI_ISL_4274026	A.21	19B
9	12/17/20	Africa / Guinea / Conakry	EPI_ISL_4274028	A.21	19B
10	12/17/20	Africa / Guinea / Kindia Region	EPI_ISL_4274030	B.1	20A
11	12/17/20	Africa / Guinea / Conakry	EPI_ISL_4274032	B.1	20A
12	2/17/21	Africa / Guinea / Nzérékoré Region	EPI_ISL_4274034	B.1.1.7	20I (Alpha)
13	2/17/21	Africa / Guinea / Conakry	EPI_ISL_4274036	R.1	20B
14	2/12/21	Africa / Guinea / Conakry	EPI_ISL_4274038	R.1	20B
15	2/12/21	Africa / Guinea / Conakry	EPI_ISL_4274040	R.1	20B
16	2/5/21	Africa / Guinea / Conakry	EPI_ISL_4274042	B.1.1.7	20I (Alpha)
17	2/5/21	Africa / Guinea / Kindia Region	EPI_ISL_4274045	B.1.1.7	20I (Alpha)
18	2/3/21	Africa / Guinea	EPI_ISL_4274047	B.1.1.7	20I (Alpha)
19	1/26/21	Africa / Guinea / Labé Region	EPI_ISL_4274049	R.1	20B

### Phylogenetic analysis

Genome sequences were aligned using MAFFT v7.453 software [18] and manually edited using MEGAX v10.1.8 [19]. All Maximum Likelihood (ML) phylogenies were inferred with IQ-Tree v2.0.5 [20] using its Model Finder function [21] before each analysis to estimate the nucleotide substitution model best-fitted for each dataset by means of Bayesian information criterion (BIC). We assessed the tree topology for each phylogeny both with the Shimodaira–Hasegawa approximate likelihood-ratio test (SH-aLRT) [22] and with ultrafast bootstrap (UFboot) [23] with 1000 replicates each. TreeTime v0.7.6 [24] was used for the assessment of root-to-tip correlation, the estimation of time-scaled phylogenies and ancestral reconstruction of most likely sequences of internal nodes of the tree and transitions between geographical locations along branches. Tree-Time was run using an autocorrelated molecular clock under a skyline coalescent tree prior. We used the sampling dates of the sequences to estimate the evolutionary rates and determine the best rooting of the tree using root-to-tip regression with least-squares method. To compare the viral distribution in Guinea to other African countries, we generated ML phylogenies combining our Guinea sequences with high-quality (high coverage and low N number) African sequences from the GISAID database. To this end, we randomly sampled 100 sequences per month from all African countries, except for South Africa (S1 Table). In the case of South Africa sequences, we randomly selected 10 per month due to the much higher number of sequences coming from this country compared to the rest of Africa. For Pango lineage distribution comparison we selected all available lineage data with complete collection date from GISAID until July 31^st^ 2021 and selected data from Guinea and the following African regions: North Africa (Algeria, Egypt, Libya, Morocco, Sudan, Tunisia, and Western Sahara), West Africa (Benin, Burkina Faso, Cape Verde, The Gambia, Ghana, Guinea-Bissau, Ivory Coast, Liberia, Mali, Mauritania, Niger, Nigeria, Senegal, Sierra Leone, and Togo), and Southern Africa (Botswana, Lesotho, Namibia, South Africa, and Swaziland).

### Statistical analysis

All statistical analyses were performed in R v4.20. We performed descriptive statistics analysis for study population characteristics using psych v2.2.5 packages comparing age, sex, symptoms, comorbidities, SARS-CoV-2 genotype and administered therapies. We compared lineage distribution by matching each lineage frequency attained from GISAID database per month and region for months when Guinea data was available and calculating spearman correlation between Guinea and the other African regions.

## Results

### Socio-demographic and clinical characteristics

The study population (n = 99 participants consented) was composed of patients with a median age of 30 ± 13.83 years (range [16–74], IQR [25–39]) and skewed male (68% male, 32% female) (Table 2). More than half (54.5%) of the study population reported between one to five contact persons at the time of symptom onset with some participants reporting more than 5 (25.3%). Dry cough (25.3%), fever (20.2%), fatigue (19.2%), and headache (18%) were the most commonly reported symptoms among participants. A small proportion of study participants reported co-morbidities, including renal failure (4.0%), liver disease (3.0%), diabetes (2.0%) and high blood pressure (2.0%). All participants who reported receiving treatment were administered a combination of chloroquine, azithromycin, vitamin C, and paracetamol as per recommended COVID-19 treatment guidelines in Guinea [25], occasionally with additional medications for symptom alleviation. All participants in this study survived the infection and none required hospitalization.

**Table 2:** General characteristics of the study population (n = 99 participants).

Characteristics	N (%)
**Sex**	
Male	67 (67.7%)
Female	32 (32.3%)
**Age Range**	
16 to 40	76 (76.8%)
41 to 60	14 (14.1%)
61 to 81	9 (9.1%)
**Contact Persons**	
1 to 5	54 (54.5%)
6 to 10	18 (18.2%)
> = 11	7 (7.1%)
Unknown/Not reported	20 (20.2%)
**First Symptom (Day)**	
1 to 7	24 (24.2%)
8 to 15	5 (5.1%)
> = 16	0 (0%)
Unknown/Not reported	70 (70.7%)
**Symptoms**	
Dry Cough	25 (25.3%)
Fever	20 (20.2%)
Fatigue	19 (19.2%)
Headache	18 (18.2%)
**Comorbidity**	
Renal Failure	4 (4.0%)
Liver disease	3 (3.0%)
Type I diabetes	2 (2.0%)
High Blood Pressure	2 (2.0%)
**Therapy**	
Chloroquine, azithromycin, vitamin C, paracetamol	93 (94.0%)
Chloroquine, azithromycin, vitamin C, paracetamol, and Other Medicine	3 (3.0%)
Unknown/Not reported	3 (3.0%)
**COVID-19 Outcome**	
Survival	99 (100%)

### Dynamics of SARS-CoV-2 strains during the first year in Guinea

**Fig 1:**
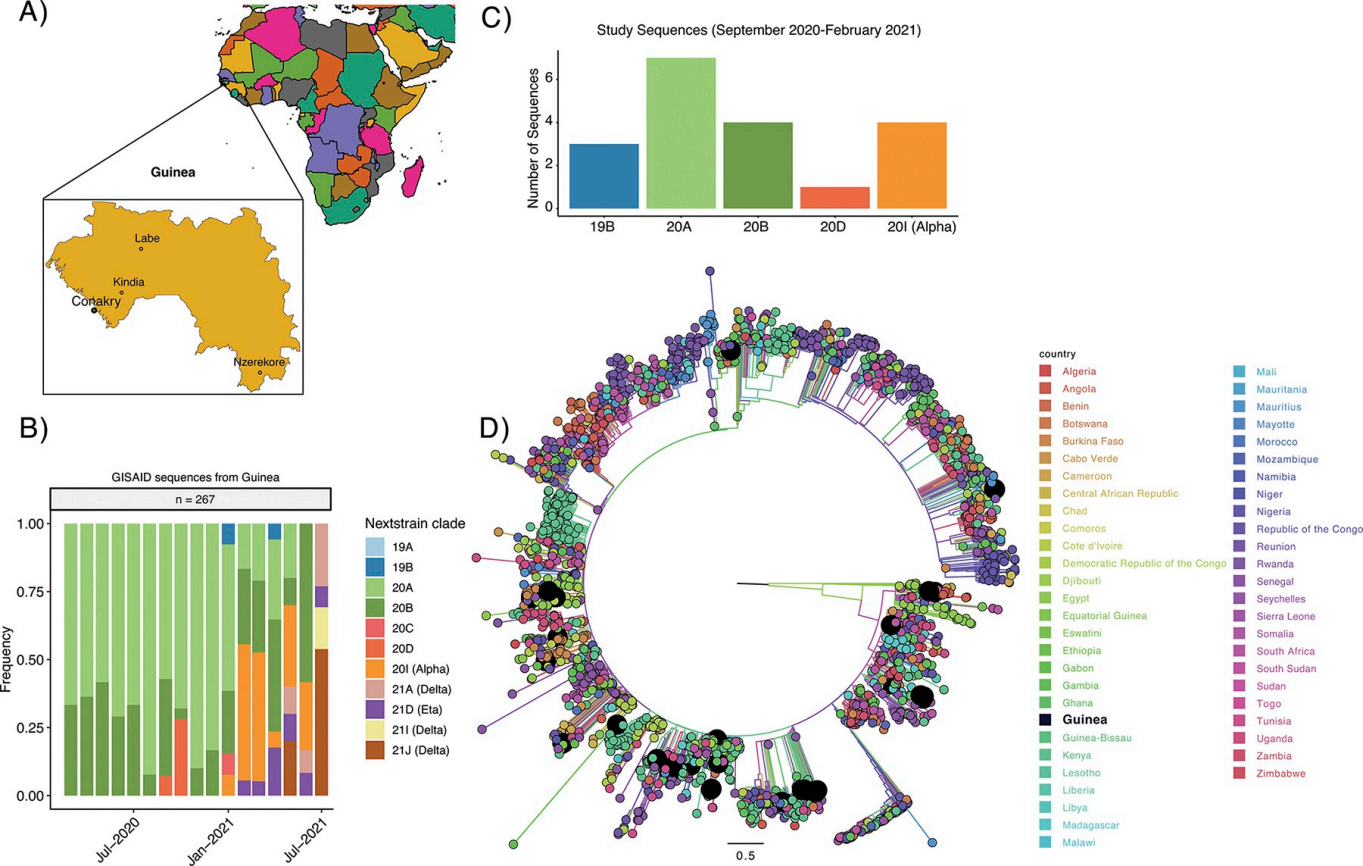
Phylogenetic analysis of SARS-CoV-2 in Guinea. A) Map of Guinea in the context of Africa indicating the cities where sequenced samples were collected. B) Distribution of the different SARS-CoV-2 clades in our sample set using Nextclade nomenclature. C) Monthly clade frequency in Guinea using all publicly available sequences from Guinea in the GISAID database until July 2021. D) ML phylogenetic temporal reconstruction of full genome sequences from all full genome sequences from this study and temporally subsampled African sequences from GISAID through July 2021. Taxa corresponding to Guinean sequences are highlighted. Branches and tips are colored by country.

Of the 99 specimens collected for genomic surveillance, complete SARS-CoV-2 whole genome sequences were able to be determined in 19 (Table 1). These 19 samples were mainly collected in Conakry, Guinea’s capital city, but we were also able to collect a small representation of other regions of the country including Kindia (n = 2), Nzérékoré (n = 1), and Labé (n = 1) (Fig 1A). Sequence analysis revealed several different clades of SARS-CoV-2 circulating in Guinea from September 2020 through February 2021, including 19B (A.21 or A.27), 20A (B.1), 20B (R.1), 20D (B.1.1.1), and 20I (B.1.1.7 or the Alpha VOC) (Fig 1B). To compare these results to those of other sequencing efforts in the region, all sequences in the GISAID database from Guinea between March 2020 and July 2021 (n = 267, including the 19 above) were downloaded and used to calculate cumulative clade frequencies by month (Fig 1C). Our dataset is largely consistent with the overall patterns of diversity observed in the rest of the database. The SARS-CoV-2 pandemic in Guinea was largely dominated by clade 20A and 20B throughout 2020. Interestingly, September and October of 2020 also have a high prevalence of clade 20D (B.1.1.1), peaking at above 25% frequency despite never reaching more than 2% globally. Subsequently, in January of 2021, we see the emergence of the Alpha VOC in the region, followed by the introduction of the 21D clade (Eta VOI) that was highly prevalent in West Africa at this time [26–28]. The Delta variant emerged in May of 2021 and became dominant by July (Fig 1D).

Interestingly, we observed a relatively high prevalence of clade 19B in our dataset despite its very low prevalence at the global scale at the time of sampling (<1% globally in December 2020) (Fig 1B–1D). Moreover, these 19B viruses had a number of unique mutations in Spike, including L452R, D614N, and N501Y. The L452R mutation appeared in all 19B sequences we reported, though it was found in only about 5% of sequences from that time period. This mutation later appeared in multiple VOIs and VOCs and has been shown to display important phenotypic properties including antibody escape [29, 30], cellular immunity evasion [31], increased affinity to ACE2 [31, 32], and increased viral shedding [29, 30, 33]. The D614N mutation appeared in two of the 19B viruses and is located in the same position as the D614G mutation that led the first SARS-CoV-2 genetic sweep [34, 35]. Finally, one of the 19B viruses had the mutation N501Y that has appeared in multiple VOCs and is thought to increase ACE2 binding affinity [36].

### Comparative frequency of SARS-CoV-2 with other African regions

**Fig 2:**
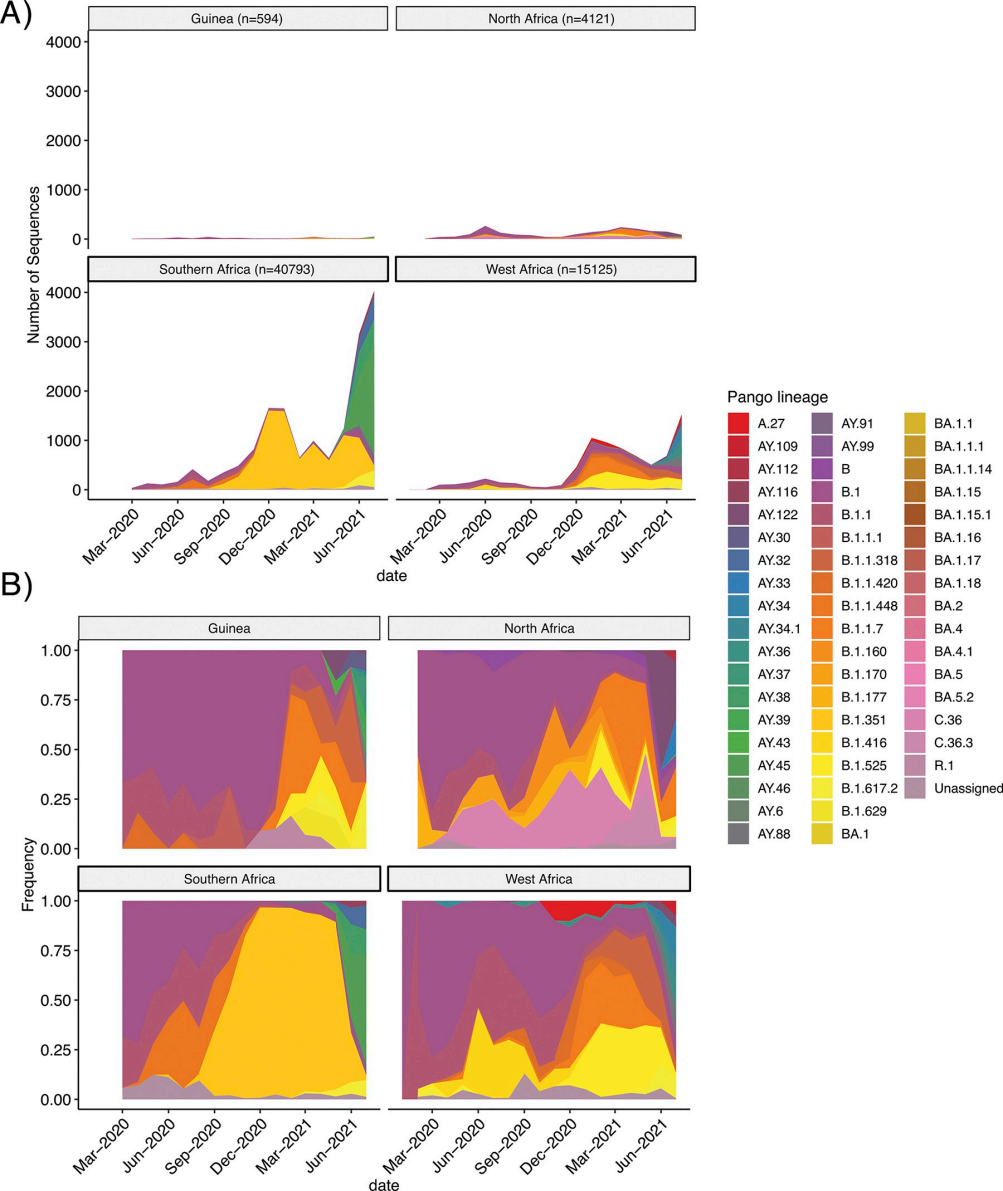
SARS-CoV-2 sequence abundance and lineage distributions among African regions in GISAID. A) Monthly number of sequences by lineage and B) monthly lineage frequency distribution using Pango nomenclature for lineages in Guinea compared to the lineages with a frequency >1% observed in surrounding African regions. All GISAID sequences deposited up to July 31st were used to examine the similarity in lineage distribution between Guinea and close by African regions. The number of sequences used per geographical location is indicated in panel A).

With the exception of South Africa, which had deposited 20,004 quality filtered SARS-CoV-2 genomes into GISAID through July 2021 [37], a majority of African countries are underrepresented in GISAID over this time period. West African countries had only submitted 15,125 SARS-CoV-2 genome sequences in GISAID, 594 of which came from Guinea (Fig 2A). At the time we initiated this study in September 2020, Guinea did not have any sequences deposited in publicly available databases despite more than 10,000 confirmed cases. To date, SARS-CoV-2 sequences originating from Africa represent only about 1.03% of the entire database. To better understand how the SARS-CoV-2 lineage distribution in 2020 and 2021 compared to other regions in Africa, we downloaded all available GISAID sequences from 2020 through July of 2021 originating from Guinea, Western Africa (other than Guinea), Northern Africa, or Southern Africa (Fig 2A). Comparing the cumulative distribution of Pango lineages [38, 39] over time (Fig 2B), Guinea was most similar to Northern African countries (rho = 0.87) than to other Western African countries (rho = 0.81) or Southern African countries (rho = 0.79). While unexpected, this may be due to Guinea’s high flight connectivity with Northern African countries, such as Morocco and Tunisia, or it may be a false association due to low sampling in the country overall.

## Discussion

This study describes SARS-CoV-2 lineage distribution during the period from October 2020 to August 2021. Despite our small sample size, we could detect relatively high levels of viral diversity in Guinea and significantly distinct viral distribution compared to other regions in Africa. Our results indicate that clade 20A was initially the most predominant clade during the last three months of 2020, followed by 20B, 19B and 20D. After January 2021, the 20I/Alpha clade became the dominant lineage in the country as was observed in other regions of the world, although its frequency in Guinea never reached levels above 60%. This is consistent with other studies that report lower frequency of Alpha in African countries compared to what was observed in Europe and North America [28]. Furthermore, we observe a very similar clade distribution to a previously reported study conducted during an overlapping time period in Guinea [40] indicating the robustness of our results.

Our study population was overall representative of most SARS-CoV-2 patients. More than half of the infected individuals (76.77%) were less than 40 years of age, the age range with the highest infection rates throughout the pandemic. Out of the patients who had documented symptom onset times, 24.2% experienced symptoms within the week leading up to their COVID-19 test. Cough, fever, and weakness were the most common symptoms reported by participants consistent with several other studies [41–43]. Altogether, our demographic and clinical data indicates that our results reflect the general characteristics of the viral dynamics ongoing in the general population and are not biased by oversampling specific populations.

A surprising observation from our analysis is the persistence of viruses from the 19B clade that according to our sequencing was circulating in Guinea in December 2020 and using publicly available sequences it could be detected in Guinea as late as April 2021 (Fig 1B), when it was extremely rare elsewhere. The clade 19B together with 19A are called early outbreak clades and were the first described clades in Wuhan, China [44, 45]. The persistence of 19B viruses in Guinea suggests that this region might have constituted a niche for this ancestral clade possibly due to lower introduction rates of more transmissible lineages, although other explanations such as specific demographic or genetic characteristics of the population in Guinea. Furthermore, these 19B viruses had a number of unique mutations in Spike that have been associated with phenotypic changes in the virus and have been included amongst VOC-defining mutations later in the pandemic, such as L452R, D614N, and N501Y. This could be indicative of convergent evolution observed in these ancestral lineages and might explain in part their persistence in the region, as some of these mutations might increase viral fitness.

As a more general observation, our results suggest a previously unreported high degree of viral variability and distinct SARS-CoV-2 dynamics that might have gone unnoticed due to considerable under sampling by molecular surveillance in countries such as Guinea. The number of sequences originating from Guinea is significantly less than the mean among other countries in West Africa, an already highly under-represented region in the genomic databases with less than 1% of the publicly available sequences. This is due to the lack of infrastructure within the country and suggests a need for networking, resources and capacity sharing in Africa [1, 46, 47]. Despite this lack of infrastructure, African countries were able to detect most of the main variants that have circulated within the continent during the pandemic, including the very early detection of Omicron lineages in Southern Africa that later dominated the global pandemic. However, this and other studies suggest that the distribution of viral lineages and therefore the viral dynamics in regions such as West Africa remain poorly understood. This can be highly significant specially given that molecular surveillance efforts are progressively decreasing globally as the COVID-19 pandemic wanes and areas that have previously constituted niches for uncommon viral lineages could be the sources of further viral diversification and appearance of new viral lineages with increased potential for immune escape and/or higher transmissibility. This suggests the need for adaptive and global systems of molecular surveillance for SARS-CoV-2 and other emerging and re-emerging pathogens in order to have successful epidemic prevention systems that are globally effective.

This project had a number of limitations. It was particularly challenging in terms of sample collection and sequencing, especially due to challenges during transportation that significantly decreased the quality of the genetic material of the samples. These are also challenges that will need improvement, most likely through development of local infrastructure to perform molecular surveillance on-site. Despite these shortcomings, our results are important as they provide a description of variants that have circulated in Guinea that indicate distinct viral dynamics in the region that will need to be considered for current and future public health epidemic prevention strategies [48, 49]. The study highlights the need for improved molecular surveillance of SARS-CoV-2 and other pathogens in African countries to adapt public health strategies within the countries and the continent [50–55].

## Conclusion

This study gives an overview of the SARS-CoV-2 lineage dynamics in Guinea from the start of the COVID-19 pandemic until mid-2021. It showed an unexpected high degree of variability and a distinct viral lineage distribution when compared to other African regions. This indicates that at least during the first phases of the SARS-CoV-2 pandemic, Guinea had limited viral introduction events, locally distinct viral circulation, and low levels of viral transmission to and/or from neighboring countries. Well-designed prospective and longitudinal studies are of need to better follow the dynamic of SARS-CoV-2 variation in the country that may provide measures for prevention, control, intervention, and treatment of this unpreceded global health scourge.

## Supporting information

S1 Table Sequences used from GISAID and acknowledgments. (XLSX)
